# Supplementary material for: Prognostic value of myocardial perfusion imaging by cadmium zinc telluride single-photon emission computed tomography in patients with suspected or known coronary artery disease: a systematic review and meta-analysis
Source: Eur J Nucl Med Mol Imaging. 2023 Jul 22;50(12):3647–58. doi: 10.1007/s00259-023-06344-8 (PMC10547640; doi:10.1007/s00259-023-06344-8)
Supplement: Supplementary file 1 — Supplementary file1 (PDF 79 KB) [file 259_2023_6344_MOESM1_ESM.pdf]

Detailed PubMed search strategy

("single-photon emission tomography" [All fields] OR "SPECT" [All fields] OR "myocardial perfusion imaging" OR "MPI" [All fields] OR "myocardial perfusion scintigraphy" OR "MPS" AND ("CZT camera" [MeSH terms] OR "cadmium-zinc telluride camera" [MeSH terms] OR "state-solid camera" [MeSH terms]) AND ("adverse outcome" [All fields] OR "follow-up" [All fields] OR "risk stratification" [MeSH terms] OR "clinical outcome" [All fields] OR "prognostic value" [MeSH terms] OR "prognosis" [MeSH terms]) AND ("coronary artery disease" [All fields] OR "CAD" [All fields]) AND ("hazard ratio" [All fields] OR "HR" [All fields])
