# Supplementary material for: Prognostic value of myocardial perfusion imaging by cadmium zinc telluride single-photon emission computed tomography in patients with suspected or known coronary artery disease: a systematic review and meta-analysis
Source: Eur J Nucl Med Mol Imaging. 2023 Jul 22;50(12):3647–58. doi: 10.1007/s00259-023-06344-8 (PMC10547640; doi:10.1007/s00259-023-06344-8)
Supplement: Supplementary file 2 — Supplementary file2 (PDF 62 KB) [file 259_2023_6344_MOESM2_ESM.pdf]

Supplementary References

1. Aburadani I, Usuda K, Sumiya H, Sakagami S, Kiyokawa H, Matsuo S, Takamura M, Murai H, Takashima S, Kitano T, Okuda K, Nakajima K. Ability of the prognostic model of J-ACCESS study to predict cardiac events in a clinical setting: The APPROACH study. *J Cardiol.* 2018;72:81-86.
2. Abuzeid W, Iwanochko RM, Wang X, Kim SJ, Husain M, Lee DS. Prognostic impact of SPECT-MPI after renal transplantation. *J Nucl Cardiol.* 2017;24:295-303.
3. Ahmed AI, Han Y, Al Rifai M, Alnabelsi T, Nabi F, Chang SM, Chamsi-Pasha MA, Nasir K, Mahmarian JJ, Cainzos-Achirica M, Al-Mallah MH. Added prognostic value of plaque burden to computed tomography angiography and myocardial perfusion imaging. *Atherosclerosis.* 2021;334:9-16.
4. Andrade LF, Souza AC, Peclat T, Bartholo C, Pavanelo T, Lima RSL. The Prognostic Value and Clinical Use of Myocardial Perfusion Scintigraphy in Asymptomatic Patients after Percutaneous Coronary Intervention. *Arq Bras Cardiol.* 2018;111:784-793.
5. Arbit B, Azarbal B, Hayes SW, Gransar H, Germano G, Friedman JD, Thomson L, Berman DS. Prognostic Contribution of Exercise Capacity, Heart Rate Recovery, Chronotropic Incompetence, and Myocardial Perfusion Single-Photon Emission Computerized Tomography in the Prediction of Cardiac Death and All-Cause Mortality. *Am J Cardiol.* 2015;116:1678-84.
6. Barros MV, Nunes Mdo C, Braga G, Rabelo DR, Generoso AB, Lima BG, Pereira LD, Prado TV, Siqueira MH. Prognostic value of coronary computed tomography in patients with non-significant perfusion defects by myocardial perfusion SPECT. *Acta Cardiol.* 2015;70:647-52.
7. Bautz J, Stypmann J, Reiermann S, Pavenstädt HJ, Suwelack B, Stegger L, Rahbar K, Reuter S, Schäfers M. Prognostic implication of myocardial perfusion and contractile

reserve in end-stage renal disease: A direct comparison of myocardial perfusion scintigraphy and dobutamine stress echocardiography. *J Nucl Cardiol.* 2022;29:2988-2999.

8. Bhatti S, Hakeem A, Dhanalakota S, Palani G, Husain Z, Jacobsen G, Ananthasubramaniam K. Prognostic value of regadenoson myocardial single-photon emission computed tomography in patients with different degrees of renal dysfunction. *Eur Heart J Cardiovasc Imaging.* 2014;15:933-40.
9. Boiten HJ, van Domburg RT, Valkema R, Schinkel AF. Eleven-year prognostic value of dobutamine stress (99m)Tc-sestamibi myocardial perfusion imaging in patients with limited exercise capacity. *Am J Cardiol.* 2015;115:884-9.
10. Boiten HJ, van Domburg RT, Valkema R, Zijlstra F, Schinkel AF. Dobutamine stress myocardial perfusion imaging: 8-year outcomes in patients with diabetes mellitus. *Eur Heart J Cardiovasc Imaging.* 2016;17:871-6.
11. Boiten HJ, van Domburg RT, Geleijnse ML, Valkema R, Zijlstra F, Schinkel AFL. Cardiac stress imaging for the prediction of very long-term outcomes: Dobutamine stress echocardiography or dobutamine 99mTc-sestamibi SPECT? *J Nucl Cardiol.* 2018;25:471-479.
12. Bom MJ, Manders JM, Uijlings R, Badings EA, Martens FM. Negative predictive value of SPECT for the occurrence of MACE in a medium-sized clinic in the Netherlands. *Neth Heart J.* 2014;22:151-7.
13. Bourantas CV, Zhang YJ, Garg S, Mack M, Dawkins KD, Kappetein AP, Mohr FW, Colombo A, Holmes DR, Ståhle E, Feldman T, Morice MC, de Vries T, Morel MA, Serruys PW. Prognostic implications of severe coronary calcification in patients undergoing coronary artery bypass surgery: an analysis of the SYNTAX study. *Catheter Cardiovasc Interv.* 2015;85:199-206.

14. Chavoshi M, Fard-Esfahani A, Fallahi B, Emami-Ardekani A, Beiki D, Hassanzadeh-Rad A, Eftekhari M. Assessment of prognostic value of semiquantitative parameters on gated single photon emission computed tomography myocardial perfusion scintigraphy in a large middle eastern population. *Indian J Nucl Med.* 2015;30:233-8.
15. Chin CT, Gao F, Keng FY, Shah BR, Koh AS, Tan RS, Chua TS. Single-photon emission computed tomography myocardial perfusion imaging-assessed stress perfusion defect severity is associated with mortality independent of ethnicity in an Asian population. *J Nucl Cardiol.* 2014;21:1148-57.
16. Djaileb L, Seiller A, Canu M, De Leiris N, Martin A, Poujol J, Fraguas-Rubio A, Leenhardt J, Carabelli A, Calizzano A, De Fondaumière M, Broisat A, Desvignes M, Vanzetto G, Ghezzi C, Fagret D, Riou LM, Barone-Rochette G. Prognostic value of SPECT myocardial perfusion entropy in high-risk type 2 diabetic patients. *Eur J Nucl Med Mol Imaging.* 2021;48:1813-1821.
17. Doukky R, Fughhi I, Campagnoli T, Wassouf M, Ali A. The prognostic value of regadenoson SPECT myocardial perfusion imaging in patients with end-stage renal disease. *J Nucl Cardiol.* 2017;24:112-118.
18. Doukky R, Nigatu A, Khan R, Anokwute C, Fughhi I, Ayoub A, Iskander F, Iskander M, Kola S, Sahyouni M, Karavolos K, Hota BN, Gomez J. Prognostic significance of ischemic electrocardiographic changes with regadenoson stress myocardial perfusion imaging. *J Nucl Cardiol.* 2020;27:1521-1532.
19. El-Hajj S, AlJaroudi WA, Farag A, Bleich S, Manaoragada P, Iskandrian AE, Hage FG. Effect of changes in perfusion defect size during serial regadenoson myocardial perfusion imaging on cardiovascular outcomes in high-risk patients. *J Nucl Cardiol.* 2016;23:101-12.

20. Engbers EM, Timmer JR, Ottervanger JP, Mouden M, Knollema S, Jager PL. Impact of Gender on the Prognostic Value of Coronary Artery Calcium in Symptomatic Patients With Normal Single-Photon Emission Computed Tomography Myocardial Perfusion. *Am J Cardiol.* 2016;118:1611-1615.

21. Engbers EM, Timmer JR, Ottervanger JP, Mouden M, Knollema S, Jager PL. Prognostic Value of Coronary Artery Calcium Scoring in Addition to Single-Photon Emission Computed Tomographic Myocardial Perfusion Imaging in Symptomatic Patients. *Circ Cardiovasc Imaging.* 2016;9:e003966.

22. Farzaneh-Far A, Shaw LK, Dunning A, Oldan JD, O'Connor CM, Borges-Neto S. Comparison of the prognostic value of regadenoson and adenosine myocardial perfusion imaging. *J Nucl Cardiol.* 2015;22:600-7.

23. Fazlinezhad, Afsoon, et al. The prognostic value of stress/rest gated myocardial perfusion SPECT in patients with known or suspected coronary artery disease. *Iranian Journal of Nuclear Medicine* 25.2 (2017): 115-121.

24. Gaibazzi N, Barbieri A, Boriani G, Benatti G, Codazzo G, Manicardi M, Bursi F, Siniscalchi C. Imaging functional stress test for stable chest pain symptoms in patients at low pretest probability of coronary artery disease: Current practice and long-term outcome. *Echocardiography.* 2019;36:1095-1102.

25. Gimelli A, Aimo A, Pasanisi EM, Coceani MA, Clemente A, Emdin M, Shaw LJ. Myocardial stress perfusion scintigraphy for outcome prediction in patients with severe left ventricular systolic dysfunction. *Eur J Nucl Med Mol Imaging.* 2021;48:3502-3511.

26. Gowdar S, Ahlberg AW, Rai M, Perucki WH, Felpel KD, Savino JA 3rd, Alter EL, Henzlova MJ, Duvall WL. Risk stratification with vasodilator stress SPECT myocardial perfusion imaging in patients with elevated cardiac biomarkers. *J Nucl Cardiol.* 2020;27:2320-2331.

27. Hage FG, Ghimire G, Lester D, Mckay J, Bleich S, El-Hajj S, Iskandrian AE. The prognostic value of regadenoson myocardial perfusion imaging. *J Nucl Cardiol.* 2015;22:1214-21.
28. Hatta T, Yoda S, Hayase M, Monno K, Hori Y, Fujito H, Suzuki Y, Matsumoto N, Okumura Y. Prognostic Value of Left Ventricular Dyssynchrony Assessed with Nuclear Cardiology in Patients with Known or Suspected Stable Coronary Artery Disease with Preserved Left Ventricular Ejection Fraction. *Int Heart J.* 2020;61:685-694.
29. Helve S, Laine M, Sinisalo J, Helanterä I, Hänninen H, Lammintausta O, Lehtonen J, Finne P, Nieminen T. Even mild reversible myocardial perfusion defects predict mortality in patients evaluated for kidney transplantation. *Eur Heart J Cardiovasc Imaging.* 2018;19:1019-1025.
30. Huang Z, Tang J, Zheng S, Jiang H, Deng L, Wang P. Prognostic significance of coronary artery calcium scoring and single-photon emission computed tomographic myocardial perfusion imaging on major adverse cardiac events in patients at low risk for suspected coronary artery disease. *Acta Cardiol.* 2019;74:508-514.
31. Huurman R, Boiten HJ, Valkema R, van Domburg RT, Schinkel AF. Eight-Year Prognostic Value of QRS Duration in Patients With Known or Suspected Coronary Artery Disease Referred for Myocardial Perfusion Imaging. *Am J Cardiol.* 2015;116:1329-33.
32. Iskander F, Iskander M, Gomez J, Doukky R. Prognostic value of regadenoson stress myocardial perfusion imaging in patients with left bundle branch block or ventricular paced rhythm. *J Nucl Cardiol.* 2021;28:967-977.
33. Kang SH, Choi HI, Kim YH, Lee EY, Ahn JM, Han S, Lee PH, Roh JH, Yun SH, Park DW, Kang SJ, Lee SW, Lee CW, Moon DH, Park SW, Park SJ. Impact of Follow-Up Ischemia on Myocardial Perfusion Single-Photon Emission Computed Tomography in Patients with Coronary Artery Disease. *Yonsei Med J.* 2017;58:934-943.

34. Kasim M, Currie GM, Tjahjono M, Siswanto BB, Harimurti GM, Kiat H. Myocardial Perfusion SPECT Utility in Predicting Cardiovascular Events Among Indonesian Diabetic Patients. *Open Cardiovasc Med J.* 2013;7:82-9.

35. Kassab K, Hussain K, Torres A, Iskander F, Iskander M, Khan R, Doukky R. The diagnostic and prognostic value of near-normal perfusion or borderline ischemia on stress myocardial perfusion imaging. *J Nucl Cardiol.* 2022;29:826-835.

36. Katsikis A, Theodorakos A, Papaioannou S, Tsapaki V, Kolovou G, Drosatos A, Koutelou M. Long-term prognostic value of myocardial perfusion imaging in octogenarians able to undergo treadmill exercise stress testing. *J Nucl Cardiol.* 2014;21:1213-22.

37. Kim HL, Kim YJ, Lee SP, Park EA, Paeng JC, Kim HK, Lee W, Cho GY, Zo JH, Choi DJ, Sohn DW. Incremental prognostic value of sequential imaging of single-photon emission computed tomography and coronary computed tomography angiography in patients with suspected coronary artery disease. *Eur Heart J Cardiovasc Imaging.* 2014;15:878-85.

38. Kolkailah AA, Iskander M, Iskander F, Patel PP, Khan R, Doukky R. The prognostic utility of regadenoson SPECT myocardial perfusion imaging in patients with end-stage renal disease: The largest cohort to date. *J Nucl Cardiol.* 2022;29:101-110.

39. Kuronuma K, Miller RJH, Otaki Y, Van Kriekinge SD, Diniz MA, Sharir T, Hu LH, Gransar H, Liang JX, Parekh et al. Prognostic Value of Phase Analysis for Predicting Adverse Cardiac Events Beyond Conventional Single-Photon Emission Computed Tomography Variables: Results From the REFINE SPECT Registry. *Circ Cardiovasc Imaging.* 2021;14:e012386.

40. Lester D, El-Hajj S, Farag AA, Bhambhvani P, Tauxe L, Heo J, Iskandrian AE, Hage FG. Prognostic value of transient ischemic dilation with regadenoson myocardial perfusion imaging. *J Nucl Cardiol.* 2016;23:1147-1155.

41. Lima RSL, Peclat TR, Souza ACAH, Nakamoto AMK, Neves FM, Souza VF, Glerian LB, De Lorenzo A. Prognostic value of a faster, low-radiation myocardial perfusion SPECT protocol in a CZT camera. *Int J Cardiovasc Imaging.* 2017;33:2049-2056.

42. Moody WE, Lin EL, Stoodley M, McNulty D, Thomson LE, Berman DS, Edwards NC, Holloway B, Ferro CJ, Townend JN, Steeds RP; Birmingham Cardio-Renal Group. Prognostic Utility of Calcium Scoring as an Adjunct to Stress Myocardial Perfusion Scintigraphy in End-Stage Renal Disease. *Am J Cardiol.* 2016;117:1387-96.

43. Moody WE, Holloway B, Arumugam P, Gill S, Wahid YS, Boivin CM, Thomson LE, Berman DS, Armstrong MJ, Ferguson J, Steeds RP. Prognostic value of coronary risk factors, exercise capacity and single photon emission computed tomography in liver transplantation candidates: A 5-year follow-up study. *J Nucl Cardiol*. 2021;28:2876-2891.

44. Nakamura S, Kawano Y, Nakajima K, Hase H, Joki N, Hatta T, Nishimura S, Moroi M, Nakagawa S, Kasai T, et al. Prognostic study of cardiac events in Japanese patients with chronic kidney disease using ECG-gated myocardial Perfusion imaging: Final 3-year report of the J-ACCESS 3 study. *J Nucl Cardiol.* 2019;26:431-440.

45. Oldan JD, Shaw LK, Hofmann P, Phelan M, Nelson J, Pagnanelli R, Borges-Neto S. Prognostic value of the cadmium-zinc-telluride camera: A comparison with a conventional (Anger) camera. *J Nucl Cardiol.* 2016;23:1280-1287.

46. Ottenhof MJ, Wai MC, Boiten HJ, Korbee RS, Valkema R, van Domburg RT, Schinkel AF. 12-Year outcome after normal myocardial perfusion SPECT in patients with known coronary artery disease. *J Nucl Cardiol.* 2013;20:748-54.

47. Park GH, Song JW, Lee CM, Song YR, Kim SG, Kim HJ, Kim JK. Long-term prognosis of end-stage renal disease patients with normal myocardial perfusion as determined by single photon emission computed tomography. *Korean J Intern Med.* 2018;33:148-156.

48. Poulin MF, Alexander S, Doukky R. Prognostic implications of stress modality on mortality risk and cause of death in patients undergoing office-based SPECT myocardial perfusion imaging. *J Nucl Cardiol*. 2016;23:202-11.

49. Roest S, Boiten HJ, van Domburg RT, Valkema R, Schinkel AFL. Prediction of 14-year cardiovascular outcomes by dobutamine stress 99mTc-tetrofosmin myocardial perfusion SPECT in elderly patients unable to perform exercise testing. *J Nucl Cardiol.* 2018;25:63-71.

50. Romero-Farina G, Candell-Riera J, Aguadé-Bruix S, Cuberas-Borrós G, Ferreira-González I, Nazarena Pizzi M, de León G, Santos A, García-Dorado D. Predictive variables for hard cardiac events and coronary revascularization in patients with normal left ventricular myocardial perfusion and systolic function. *Eur J Nucl Med Mol Imaging.* 2013;40:1181-9.

51. Smith P, Farag A, Bhambhvani P, Iskandrian A, Hage FG. Prognostic value of absent left ventricular ejection fraction reserve with regadenoson SPECT MPI. *J Nucl Cardiol.* 2022;29:978-986.

52. Songy B, Guernou M, Hivoux D, Attias D, Lussato D, Queneau M, Bonardel G, Bertaux M. Prognostic value of one millisievert exercise myocardial perfusion imaging in patients without known coronary artery disease. *J Nucl Cardiol.* 2018;25:120-130.

53. Stochkendahl MJ, Mickley H, Vach W, Aziz A, Christensen HW, Hartvigsen J, Høilund-Carlsen PF. Clinical characteristics, myocardial perfusion deficits, and clinical outcomes of patients with non-specific chest pain hospitalized for suspected acute coronary syndrome: a 4-year prospective cohort study. *Int J Cardiol.* 2015;182:126-31.

54. Tamarappoo BK, Otaki Y, Sharir T, Hu LH, Gransar H, Einstein AJ, Fish MB, Ruddy TD, Kaufmann P, Sinusas AJ, Miller EJ, Bateman TM, Dorbala S, Di Carli M, Eisenberg E, Liang JX, Dey D, Berman DS, Slomka PJ. Differences in Prognostic Value of

Myocardial Perfusion Single-Photon Emission Computed Tomography Using High-Efficiency Solid-State Detector Between Men and Women in a Large International Multicenter Study. *Circ Cardiovasc Imaging.* 2022;15:e012741.

55. Tottleben J, Howland J, Rofael M, Co MLF, Torres A, Doukky R. The prognostic and diagnostic implications of surveillance serial myocardial perfusion imaging in asymptomatic renal transplant candidates. *J Nucl Cardiol.* 2022 Jun 15.
56. Toyama T, Kasama S, Sato M, Sano H, Ueda T, Sasaki T, Nakahara T, Higuchi T, Tsushima Y, Kurabayashi M. Most Important Prognostic Values to Predict Major Adverse Cardiovascular, Cerebrovascular, and Renal Events in Patients with Chronic Kidney Disease Including Hemodialysis for 2 Years. *Cardiology.* 2019;142:14-23.
57. Veenis JF, Valkema R, van Domburg RT, Schinkel AF. Prediction of 14-year outcomes in patients with a limited exercise capacity: Utility of dobutamine myocardial perfusion imaging in a high-risk population. *J Nucl Cardiol.* 2015;22:888-900.
58. Weintraub WS, Hartigan PM, Mancini GBJ, Teo KK, Maron DJ, Spertus JA, Chaitman BR, Shaw LJ, Berman D, Boden WE. Effect of Coronary Anatomy and Myocardial Ischemia on Long-Term Survival in Patients with Stable Ischemic Heart Disease. *Circ Cardiovasc Qual Outcomes.* 2019;12:e005079.
59. Yoda S, Nakanishi K, Tano A, Hori Y, Hayase M, Mineki T, Suzuki Y, Matsumoto N, Hirayama A. Prognostic Value of Major Cardiac Event Risk Score Estimated With Gated Myocardial Perfusion Imaging in Japanese Patients With Coronary Artery Disease. *Int Heart J.* 2016;57:408-16.
60. Yokota S, Mouden M, Ottervanger JP, Engbers E, Knollema S, Timmer JR, Jager PL. Prognostic value of normal stress-only myocardial perfusion imaging: a comparison between conventional and CZT-based SPECT. *Eur J Nucl Med Mol Imaging.* 2016;43:296-301.

61. Zellweger MJ, Fahrni G, Ritter M, Jeger RV, Wild D, Buser P, Kaiser C, Osswald S, Pfisterer ME; BASKET Investigators. Prognostic value of "routine" cardiac stress imaging 5 years after percutaneous coronary intervention: the prospective long-term observational BASKET (Basel Stent Kosteneffektivitäts Trial) LATE IMAGING study. *JACC Cardiovasc Interv*. 2014;7:615-21.
