# Supplementary material for: Prognostic value of myocardial perfusion imaging by cadmium zinc telluride single-photon emission computed tomography in patients with suspected or known coronary artery disease: a systematic review and meta-analysis
Source: Eur J Nucl Med Mol Imaging. 2023 Jul 22;50(12):3647–58. doi: 10.1007/s00259-023-06344-8 (PMC10547640; doi:10.1007/s00259-023-06344-8)
Supplement: Supplementary file 3 — Supplementary file3 (PDF 113 KB) [file 259_2023_6344_MOESM3_ESM.pdf]

Figure Legends

Figure S1. Methodological quality of the included studies assessed with QUIPS tool for risk of bias and applicability concerns. The green circle represents low risk of bias, the yellow circle unclear risk of bias and the red circle high risk of bias.

Figure S2. Funnel plot for the risk of adverse events associated with abnormal myocardial perfusion. Each dot represents a study; the y-axis represents study precision (standard error of effect size), and the x-axis shows the effect size. Large studies appear toward the top of the graph and tend to cluster near the mean effect size. Small studies appear toward the bottom of the graph and are dispersed across a range of values since there is more sampling variation in effect size estimates. The outer dashed lines indicate the triangular region within which 95% of studies are expected to lie in the absence of biases and heterogeneity. lnHR, natural logarithm of hazard ratio; S.E., standard error.

Figure S3. Funnel plot for the risk of adverse events with missing studies identified by the trim and fill method (dots: original studies; triangles: filled missing studies). The vertical solid line shows the pooled estimate including the filled studies. lnHR, natural logarithm of hazard ratio; S.E., standard error.

Figure S4. Funnel plot for the incidence rate ratio of adverse events in patients with abnormal versus normal myocardial perfusion. Each dot represents a study; the y-axis represents study precision (standard error of effect size), and the x-axis shows the effect size. Large studies appear toward the top of the graph and tend to cluster near the mean effect size. Small studies appear toward the bottom of the graph and are dispersed across a range of values since there is more sampling variation in effect size estimates. The outer dashed lines indicate the triangular region within which 95% of studies are expected to lie in the absence of both biases and heterogeneity. lnIRR, natural logarithm of incidence rate ratio; S.E., standard error.

Figure S1

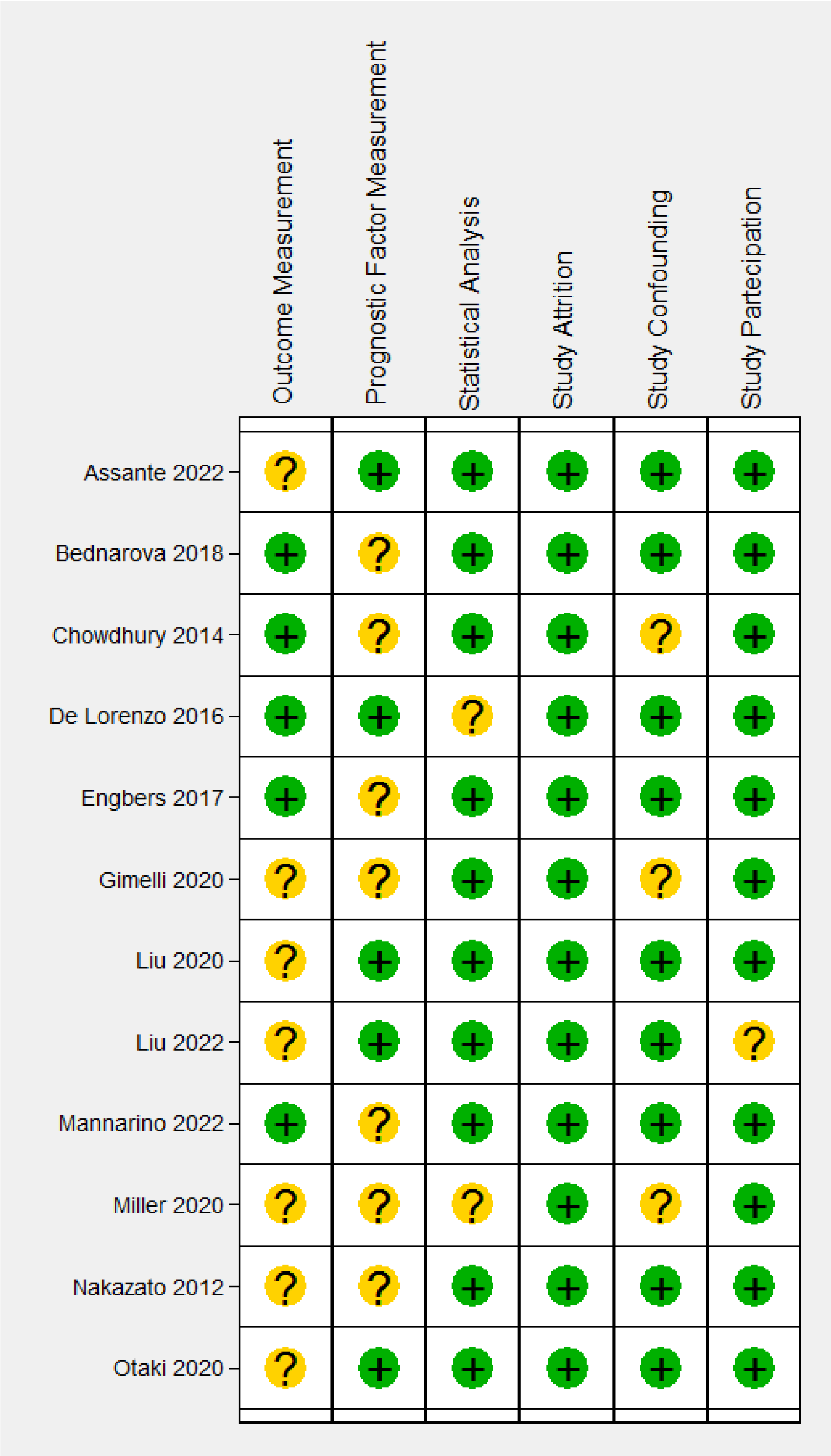
Outcome Measurement
Prognostic Factor Measurement
Statistical Analysis
Study Attrition
Study Confounding
Study Partecipation
Assante 2022
Bednarova 2018
Chowdhury 2014
De Lorenzo 2016
Engbers 2017
Gimelli 2020
Liu 2020
Liu 2022
Mannarino 2022
Miller 2020
Nakazato 2012
Otaki 2020

Figure S2

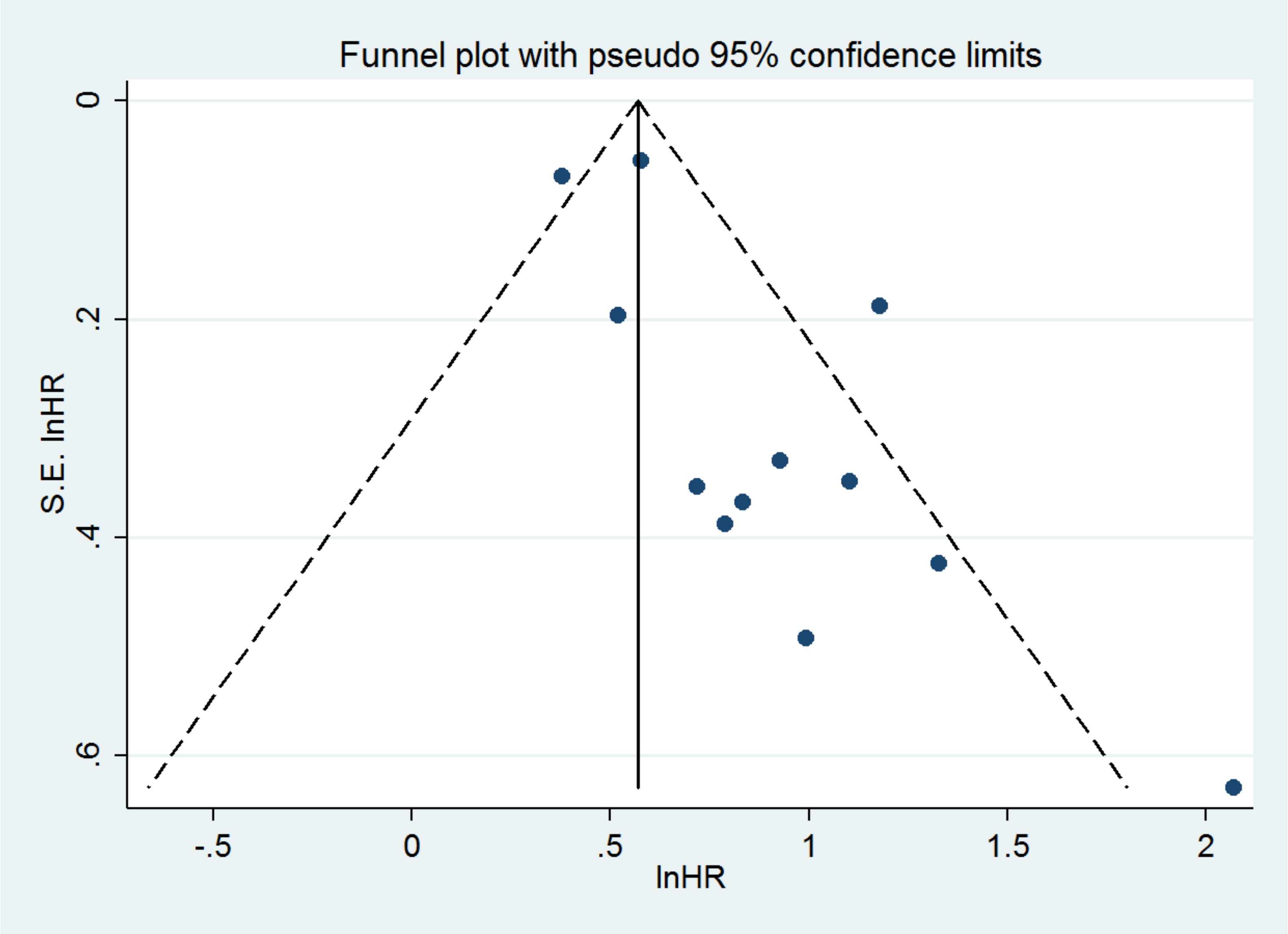
Funnel plot with pseudo 95% confidence limits
0
.2
.4
.6
S.E. lnHR
-.5
0
.5
1
1.5
2
lnHR

Figure S3

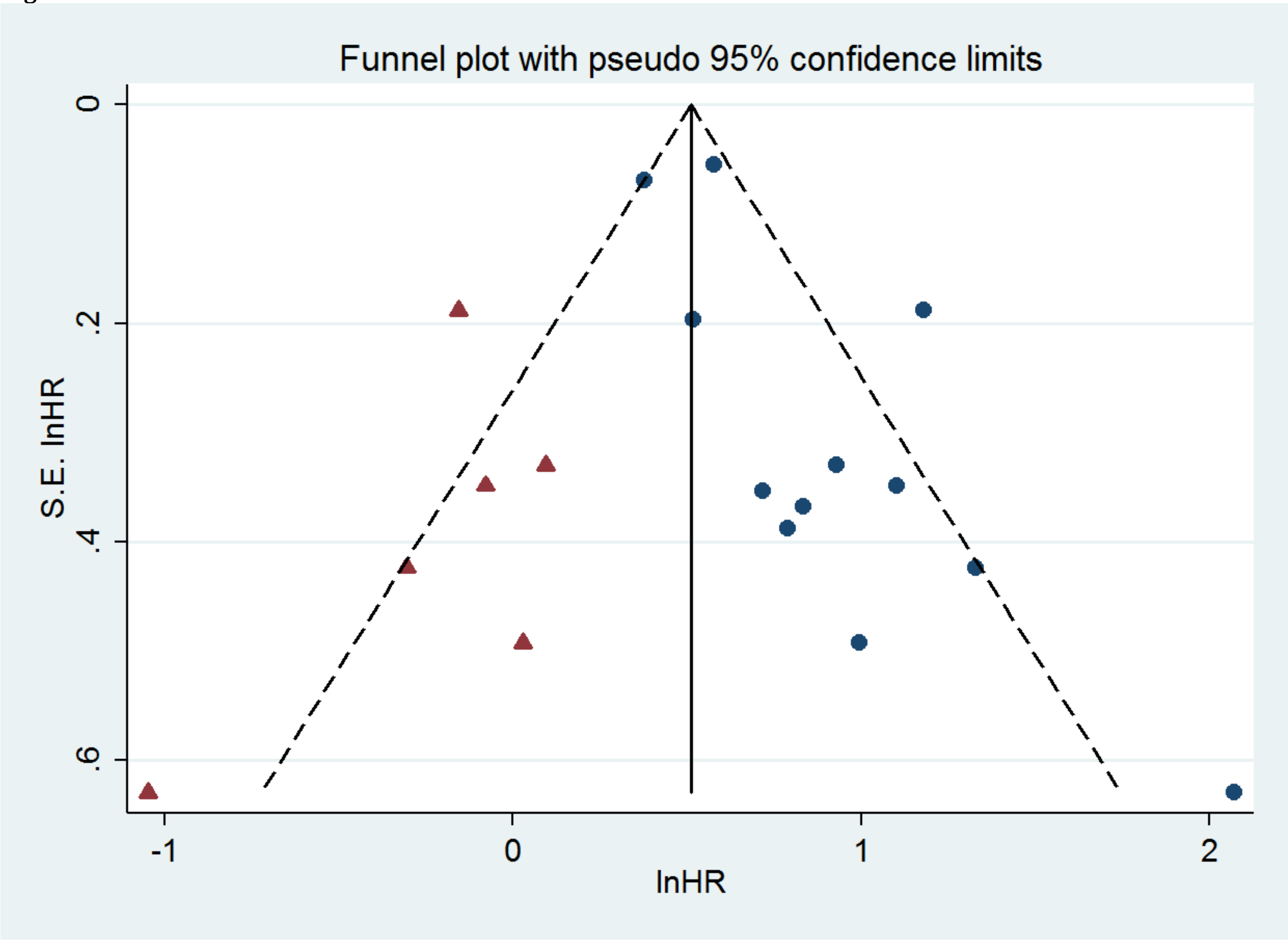

Funnel plot with pseudo 95% confidence limits
S.E. lnHR
0
.2
.4
.6
-1
0
1
2
lnHR

Figure S4

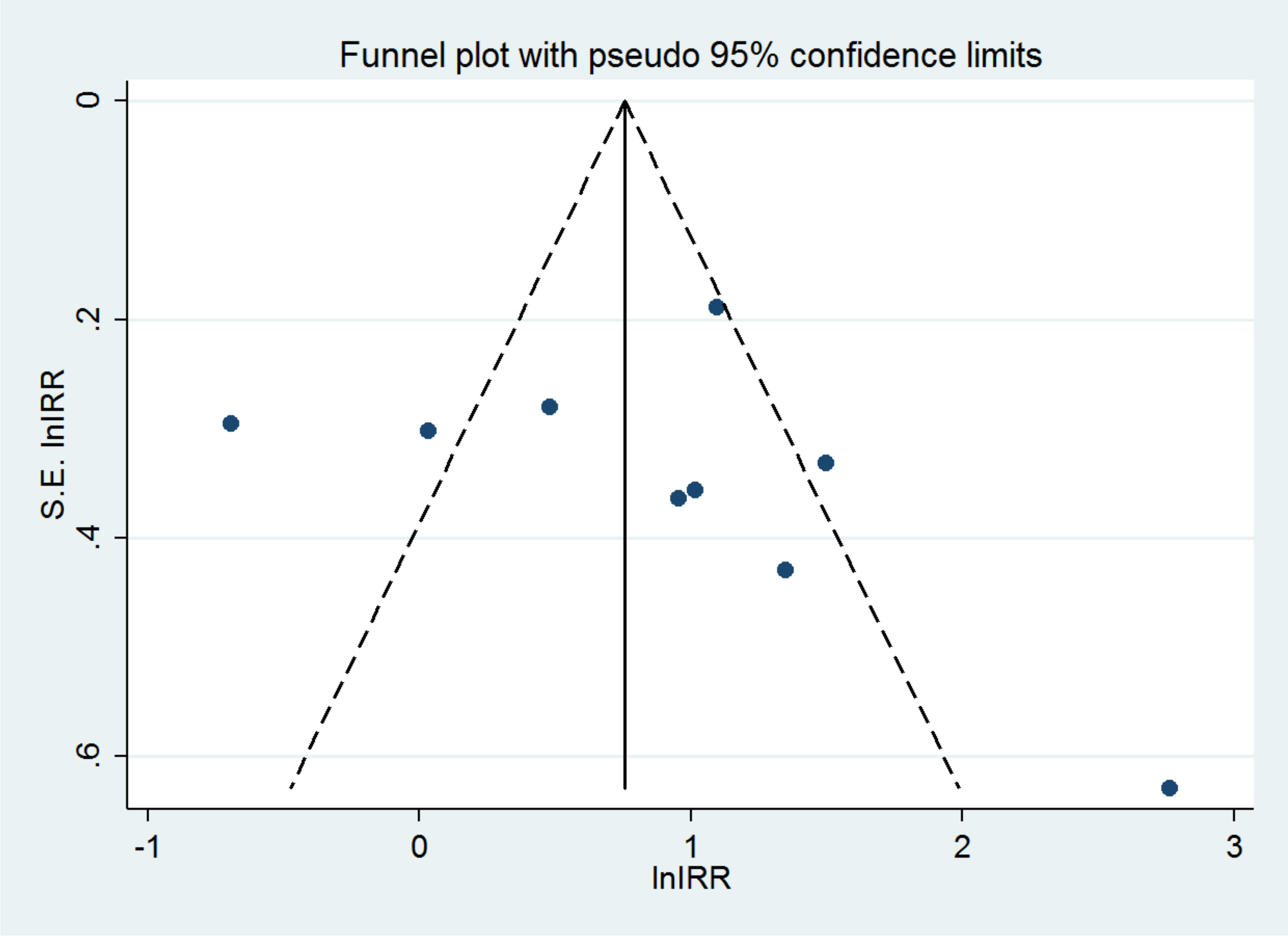

Funnel plot with pseudo 95% confidence limits
0
.2
.4
.6
S.E. lnIRR
-1
0
1
2
3
lnIRR
