# Supplementary material for: Prognostic value of myocardial perfusion imaging by cadmium zinc telluride single-photon emission computed tomography in patients with suspected or known coronary artery disease: a systematic review and meta-analysis
Source: Eur J Nucl Med Mol Imaging. 2023 Jul 22;50(12):3647–58. doi: 10.1007/s00259-023-06344-8 (PMC10547640; doi:10.1007/s00259-023-06344-8)
Supplement: Supplementary file 4 — Supplementary file4 (PDF 66 KB) [file 259_2023_6344_MOESM4_ESM.pdf]

Table S1. **Leave one-out sensitivity analysis**

Study omitted	Hazard ratio of adverse events
Assante et al. (25)	2.18 (1.77-2.68)
Bednarova et al. (26)	2.15 (1.74-2.65)
Chowdhury et al. (27)	2.07 (1.72-2.50)
De Lorenzo et al. (28)	2.17 (1.76-2.67)
Engbers et al. (29)	2.12 (1.73-2.60)
Gimelli et al. (30)	1.97 (1.64-2.36)
Liu et al. (31)	2.19 (1.77-2.70)
Liu et al. (32)	2.16 (1.75-2.65)
Mannarino et al. (33)	2.10 (1.72-2.56)
Miller et al. (34)	2.39 (1.90-3.01)
Nakazato et al. (35)	2.27 (1.82-2.83)
Otaki et al. (36)	2.40 (1.80-3.21)

Table S2. Leave one-out sensitivity analysis IRR

Study omitted	Incidence rate ratio of adverse events
Assante et al. (25)	2.36 (1.30-4.30)
Bednarova et al. (26)	2.65 (1.49-4.72)
Chowdhury et al. (27)	2.01 (1.20-3.36)
De Lorenzo et al. (28)	2.34 (1.29-4.26)
Engbers et al. (29)	2.19 (1.23-3.89)
Gimelli et al. (30)	2.33 (1.23-4.39)
Liu et al. (31)	2.52 (1.36-4.66)
Liu et al. (32)	2.81 (1.83-4.32)
Mannarino et al. (33)	2.25 (1.26-4.01)
